# Mental health disturbance in preclinical medical students and its association with screen time, sleep quality, and depression during the COVID-19 pandemic

**DOI:** 10.1186/s12888-024-05512-w

**Published:** 2024-01-31

**Authors:** Tjhin Wiguna, Valerie Josephine Dirjayanto, Zhahna Siti Maharani, Emir Gibraltar Faisal, Sylvie Dominic Teh, Erik Kinzie

**Affiliations:** 1 Faculty of Medicine Universitas Indonesia, Dr. Cipto Mangunkusumo General Hospital, Jakarta, Indonesia; 2 Faculty of Medicine, Universitas Indonesia, Jakarta, Indonesia; 3 Faculty of Medical Sciences, Newcastle University, Newcastle upon Tyne, UK; 4 Department of Psychiatry, Tulane University School of Medicine, New Orleans, USA

**Keywords:** COVID-19, Depression, Indonesia, Medical students, Mental health, Screen time, Sleep quality

## Abstract

**Background:**

Affected by various hurdles during COVID-19, preclinical medical students are at an elevated risk for mental health disturbances. However, the effects of modern mental health problems on preclinical medical students have not been adequately researched. Thus, this study was aimed to identify the proportions and implications of current mental health problems for depression, sleep quality and screen time among Indonesian medical preclinical students during the COVID-19 pandemic.

**Methods:**

This cross-sectional study was conducted using crowdsourcing between October 2020 and June 2021. During the study period, 1,335 subjects were recruited, and 1,023 datasets were identified as valid. General Health Questionnaire-12 (GHQ-12) was used to measure current mental health disturbances (categorized as without current mental health disturbances, psychological distress, social dysfunction, or both). The Patient Health Questionnaire-9 (PHQ-9) was used to assess depression, the Pittsburgh Sleep Quality Index (PSQI) was employed to assess sleep quality, and a questionnaire devised for this study was used to assess screen time length per day. Multivariate data analysis was conducted using SPSS version 24 for Mac.

**Results:**

According to the findings, 49.1% of the 1,023 participants had current mental health disturbances: 12.8% had psychological distress, 15.9% had social dysfunction, and the rest (20.4%) had both psychological distress and social dysfunction. The statistical analysis provided strong evidence of a difference (*p* < 0.001) between the medians of depression and sleep quality with at least one pair of current mental health disturbance groups, but the difference for screen time was not significant (*p* = 0.151). Dunn’s post-hoc analysis showed that groups without current mental health problems had significantly lower mean ranks of depression and sleep quality compared to groups that had current mental health problems (*p* < 0.001).

**Conclusion:**

Current mental health disturbances during the COVID-19 pandemic were significantly associated with preclinical medical students’ depression and sleep quality in preclinical medical students. Thus, mental health programs for this specific population should be tailored to integrate mindfulness therapy, support groups, stress management, and skills training to promote mental wellbeing.

## Background

The World Health Organization (WHO) includes mental health within the definition of health as a whole, which is not only the absence of disorders but also a spectrum of mental well-being that allows a person to manage daily life stressors, interact with others, exercise productivity, and successfully contribute to the community [1]. Thus, the impact of mental health on many aspects of life is vast and significant, especially when disturbances occur.

COVID-19 wreaked havoc on the global population, causing more than 545 million cases and six million deaths as of July 2022 [2]. This raised awareness about mental health as COVID-19 could result in neuropsychological repercussions both due to the disease itself and via changes within the society [3]. Between 2020 and 2021, Indonesia experienced two waves of COVID-19, with total cases reaching six million and deaths amounting to more than 156 thousand [4]. Considering Indonesia’s strong culture of kinship and gatherings, the rising number of cases and large-scale social restrictions across the country also led to concerns over mental health, especially for the most vulnerable, such as the youth. These concerns were justified, considering that previous studies discovered high prevalence of mental health problems among youths, including depression [5] and anxiety disorders [6].

Among youths, medical students, who are required to earn more credits than other majors, were at an even higher risk of mental health disturbances due to multiple additional challenges, including restricted peer engagement, uncertainties regarding exams, struggles to understand their roles, and worry about themselves and their loved ones getting infected by COVID-19 [7]. Since the medical subject is particularly demanding, worry, depression, and mental health problems during COVID-19 could pose a greater impact [8]. Son et al. demonstrated that 71% of medical students experienced elevated stress during the COVID-19 pandemic, a significantly higher number compared to 27.2% before the pandemic [9]. Moreover, medical students who stayed at home, who were in a similar state to preclinical students in Indonesia, faced higher incidences of depression than those living in hostels [10]. In Bangladesh, during COVID-19, the prevalence of severe depression among university students was 26.7% [11]. In India, undergraduate medical interns and postgraduate medical residents showed high perceived stress levels (scoring 27–40), and 69% of the individuals reported moderate stress (scoring 14–26), owing to overwhelming responsibilities and inadequate resources [12]. Mental health disturbances among medical students during the COVID-19 pandemic could trigger negative socioeconomic consequences, such as limited part time job offers and increased access to mental healthcare facilities. Thus, it is appropriate for public health interventions to consider the need for better mental health among this cohort [13].

With increasing accessibility for all socioeconomic levels, the internet has now become very influential in daily lives. This triggered a new and worrisome issue: problematic internet usage, affecting adolescents and young adults, as objectively proven with increased screen time duration [14]. A total of 5.07 billion people currently utilize the internet globally, and the latest statistics indicate that the world’s connected population rose by more than 170 million between October 2021 and December 2022 [15]. During the pandemic, the majority of screen time among medical students (51.9%) was spent on social media, which could be the manifestation of an attempt to maintain connectivity [16]. While it could yield positive effects considering its multifunctionality in entertainment, shopping, and education, screen time might also cause problematic drawbacks, even among medical students [17]. In association with increased screen time, numerous psychological problems, including social isolation, stress, and reduced concentration might arise.

Along with the rise in mental health problems, worry about getting infected with COVID-19, as identified by Taylor et al. [18] as one of the indicators of COVID-19 stress syndrome, also escalated. This worry was described as more prominent during the first weeks of the lockdown measures [19], thus causing possibly significant impairment in productivity. Moreover, studies have also suggested that an increase in mental health disturbances may trigger depression, increase screen time, and aggravate sleep disturbances during the COVID-19 pandemic. A higher cumulative screen time– associated with self-harm and decreased self-esteem [20]– could be explained by the overlap of brain regions involved, particularly the subgenual cingulate cortex (sgCC) and orbitofrontal cortex (OFC) [21]. Furthermore, sleep and emotional states might be regulated through common pathways involving the imbalance of neurotransmitters, anomalies in brain activation, disruption of the hypothalamic‒pituitary‒adrenal (HPA) axis, and inflammation [22, 23].

For medical students, vulnerability to depression, problematic use of digital media, and lack of good sleep quality due to disrupted mental well-being demand effective intervention strategies both during COVID-19 and beyond. However, as of currently, there is a lack of understanding of how these factors could be associated with each other. Depression, poor sleep quality, and increased screen time were proven to significantly worsen in previous studies [24], but reciprocal relationships between mental health disturbances might also exist, and this converse aspect was less studied [25]. Thus, this study was aimed to further clarify the association between current mental health disturbances and depression, screen time, and sleep problems among preclinical medical students during the COVID-19 pandemic. This would provide a basis for specific areas of intervention for this cohort, especially if promoting mental health was associated with corresponding improvement in depression, worry, screen time, and sleep problems.

## Methods

### Study design and data collection

This cross-sectional study was conducted using crowdsourcing and snowballing methods to help gather more participants within a short period of time. The data were collected using an online questionnaire (http://surveymonkey.com), and the survey link was distributed via local medical organizational groups across Indonesia and personal chats on social media platforms such as WhatsApp groups, Line, Facebook, and Instagram. Prior to the study, ethical approval for the research protocol was obtained from the Health Research Ethical Committee of the Faculty of Medicine at Universitas Indonesia. The study participants included preclinical medical students who underwent online learning and provided their consent through an online informed consent form. The preclinical period of study in Indonesia lasts until four and a half years, during which the students are required to study the medical sciences theoretically without having to meet face-to-face with the patients. Those who did not complete the questionnaire were excluded. The collection period was from October 2020 to June 2021. During the study period, 1,335 participants filled in the online questionnaire through the survey link, but only 1,023 datasets were filled out completely (response rate 76.6%) and were finally eligible for statistical analysis after excluding 56 duplicates and 256 incomplete sets. The respondents were preclinical medical students studying at 64 medical faculties across Indonesia from 30 out of 34 provinces in the country.

### Research instruments

The research questionnaire began with an informed consent form and consisted of several sections, the first of which aimed to determine the respondents’ sociodemographic characteristics. The instruments used in the following sections included the General Health Questionnaire (GHQ-12), Patient Health Questionnaire-9 (PHQ-9), Pittsburgh Sleep Quality Index (PSQI), and a screen time questionnaire.

### GHQ-12

The GHQ-12 was used to assess psychological distress and social dysfunction as domains of current mental health disturbances using a 12-item self-report questionnaire. It was selected to analyze the current mental health disturbances of participants within a two-week time frame, yielding a total score between 0 and 18 for the psychological distress and social dysfunction domains. A higher total score on both domains indicates more current mental health disturbances. Because the GHQ-12 has no exact cutoff point for each domain, data-driven cutoffs were used. This study found that the psychological distress and social dysfunction total scores were not distributed normally; thus, the median was used for the data-driven cutoffs. Participants with a total score of psychological distress and social dysfunction under the median (medians of less than 13 for psychological distress and less than eight for social dysfunction) were determined as not having current mental health disturbances. Participants with a median score of 13 and above for psychological distress (total score) were determined to have psychological distress, those with a median score of eight and above for social dysfunction were determined to have social dysfunction, and those who scored in the indicated ranges for both domains were determined to have both psychological distress and social dysfunction. Based on the GHQ-12 results, the study identified four groups of participants: without current mental health disturbances, with psychological distress, with social dysfunction, and with both psychological distress and social dysfunction. The GHQ-12 was translated into Indonesian and validated by Idaiani et al., with a Cronbach’s α of 0.670–0.77. Sensitivity and specificity analyses yielded values of 67.89% and 74.75%, respectively [26].

### PHQ-9

The PHQ-9 was used to evaluate depression, translated into Indonesian, and validated by van der Linden et al. with a Cronbach’s α of 0.724 [27]. The questionnaires were scored on a Likert-type scale, ranging from 0 to 3, with higher numbers indicating more severe depressive symptoms. A total score between 0 and 4 indicates no or minimal depression, 5–9 indicates mild depression, 10–14 indicates moderate depression, 15–19 indicates moderately severe depression, and 20–27 indicates severe depression [28].

### PSQI

The influence of sleep was further measured using the Indonesian version of the PSQI questionnaire, validated with a Cronbach’s α of 0.83 [29]. Using this instrument, sleep was evaluated on seven dimensions: (1) subjective sleep quality (measured as a response to question 9 regarding how the respondent would rate overall sleep quality), (2) sleep latency (sum of questions 2 and 5a regarding the duration to fall asleep and how often the respondent could not fall asleep within 30 min within the past month, respectively), (3) sleep duration (the amount of actual sleep; >7 h = 0, 6–7 h = 1, 5–6 h = 2, and < 5 h = 3), (4) sleep habitual efficiency (calculated from questions 1, 3, and 4; 0 = > 85% efficiency, 1 = 75–84%, 2 = 65–74%, and 3 = < 65%), (5) sleep disturbances (estimated via how often the respondent experienced sleeping problems), (6) use of medications (0 = none, 1 = less than once a week, 2 = once or twice a week, and 3 = 3 or more times a week), and (7) daytime activity dysfunction (sum of answers to questions 7 and 8), each scored using a Likert scale ranging from 0 to 3, with greater scores indicating greater disturbances [29, 30]. The maximum total score was 21, and the global cutoff was set at 5 (29), above which the participant was interpreted as having poor sleep quality.

### Screen-time questionnaire

Screen time was measured using a modified youth screen time questionnaire. Daily total screen time per day was calculated by summing the average weekday and weekend screen time for the four typical activities (Smart phones, laptops, television, and other screen time activities) and divided by seven. The method used to measure screen time followed standard methods applied in several peer-reviewed studies [31]. The questionnaire did not need any validation because it is asking the number of time (in minute) spent on each typical screen time activities per day on weekend and weekday.

### Statistical analysis

Data analysis was performed using IBM SPSS version 24 for Mac (IBM, Armonk, New York, USA). Normally distributed numerical variables, as revealed by the Shapiro‒Wilk test, are reported as the mean (standard deviation), while nonnormally distributed variables are reported as the median (interquartile range). Meanwhile, categorical variables are reported as numbers (percentage). To identify the effect of current mental health disturbances (as grouped by the GHQ-12) on depression, sleep quality and screen time among preclinical students during the COVID-19 pandemic, one-way ANOVA on ranks test (the Kruskal‒Wallis H test) was conducted [32]. In addition, the Dunn’s post hoc tests were carried out to determine the mean rank differences in each group. The level of significance was set at less than 0.05 (*p* < 0.05).

## Results

The participants’ study year ranged from the first to the fourth year of medical school, with most students coming from moderate- to high- income families (Table 1). The median age of all participants was 20 years (range: 17–27). The GHQ-12 results revealed that most of the participants did not have any current mental health disturbances (50.9%), while 49.1% experienced current mental health disturbances consisting of psychological distress (12.8%), social dysfunction (15.9%), or both (20.4%). Different degrees of depression were present, as follows: none to minimal (4.7%), mild (18%), moderate (35.3%), moderately severe (31.7%), and severe (10.4%). Moreover, based on the PSQI, more students experienced poor sleep quality (61%) than not (39%). Regarding daily screen time, the students spent a median of 930 min per day on all devices, with 430 min per day spent on laptops and 360 min on smartphones; however, screen times for video games and online games were less than one hour per day (Table 1).

**Table 1:** Characteristics of participants (*n* = 1023)

Characteristics	Median (Interquartile Range)	Frequency (%)
Age (year)	20 (19–20)	
Gender		
Male Female		313 (30.6) 710 (69.4)
Education Year		
First year Second year Third year Fourth year (preclinical)		162 (15.8) 419 (41.0) 327 (32.0) 112 (11.2)
Family Financial Background		
High family income level Moderate family income level Low family income level Very low family income level		427 (41.7) 362 (35.4) 195 (19.1) 39 (3.8)
Current Mental Health Disturbances (GHQ-12)	18 (15–21)	
Without current mental health disturbances Psychological distress Social dysfunction Both psychological distress and social dysfunction		521 (50.9) 131 (12.8) 163 (15.9) 208 (20.4)
Depression (PHQ-9)	13 (10–17)	
None to minimal depression Mild depression Moderate depression Moderate to severe depression Severe depression		48 (4.7) 184 (18.0) 361 (35.3) 324 (31.7) 106 (10.4)
Sleep Quality (PSQI)	6 (4–9)	
Without sleep disturbance With sleep disturbance		399 (39) 624 (61)
Total Screen Time per day (minutes) Screen time for television Screen time for laptop Screen time for smartphones Screen time for video games Screen time for online games	930 (660–1208) 10 (0–60) 430 (300–600) 360 (180–540) 0 (0–7.50) 0 (0–60)	

**Table 2:** The Dunn’s post hoc analysis for depression (PHQ-9)

Current Mental Health	Mean Rank Difference	Std. Error	Std. Deviation of Mean Rank	Sig.	Adj. Sig.
WCMH-PD	-214.827	28.819	-7.454	0.000	0.000
WCMH-SD	-310.432	26.462	-11.731	0.000	0.000
WCMH-Both	-437.012	24.183	-18.071	0.000	0.000
PD-SD	-95.604	34.598	-2.763	0.006	0.034
PD-Both	-222.185	32.888	-6.756	0.000	0.000
SD-Both	-126.581	30.844	-4.104	0.000	0.000

WCMH = without current mental health disturbances, PD = psychological distress, SD = social dysfunction, Both = with psychological distress and social dysfunction. Each row tests the null hypothesis that the group distributions are equivalent. Asymptotic significance (2-sided tests) is displayed. The significance level is set at 0.05. Significance values were adjusted by Bonferroni correction for multiple tests

**Table 3:** The Dunn’s post hoc analysis for sleep quality (PSQI)

Current Mental Health	Mean Rank Difference	Std. Error	Std. Deviation of Mean Rank	Sig.	Adj. Sig.
WCMH-PD	-102.541	28.744	-3.567	0.000	0.002
WCMH-SD	-180.188	26.394	-6.827	0.000	0.000
WCMH-Both	-260.505	24.121	-10.800	0.000	0.000
PD-SD	-77.648	34.509	-2.250	0.024	0.147
PD-Both	-157.964	32.803	-4.816	0.000	0.000
SD-Both	-80.317	30.764	-2.611	0.009	0.054

WCMH = without current mental health disturbances, PD = psychological distress, SD = social dysfunction, Both = with psychological distress and social dysfunction. Each row tests the null hypothesis that the group distributions are equivalent. Asymptotic significance (2-sided tests) is displayed. The significance level is set at 0.05. Significance values were adjusted by Bonferroni correction for multiple tests

**Fig. 1:**
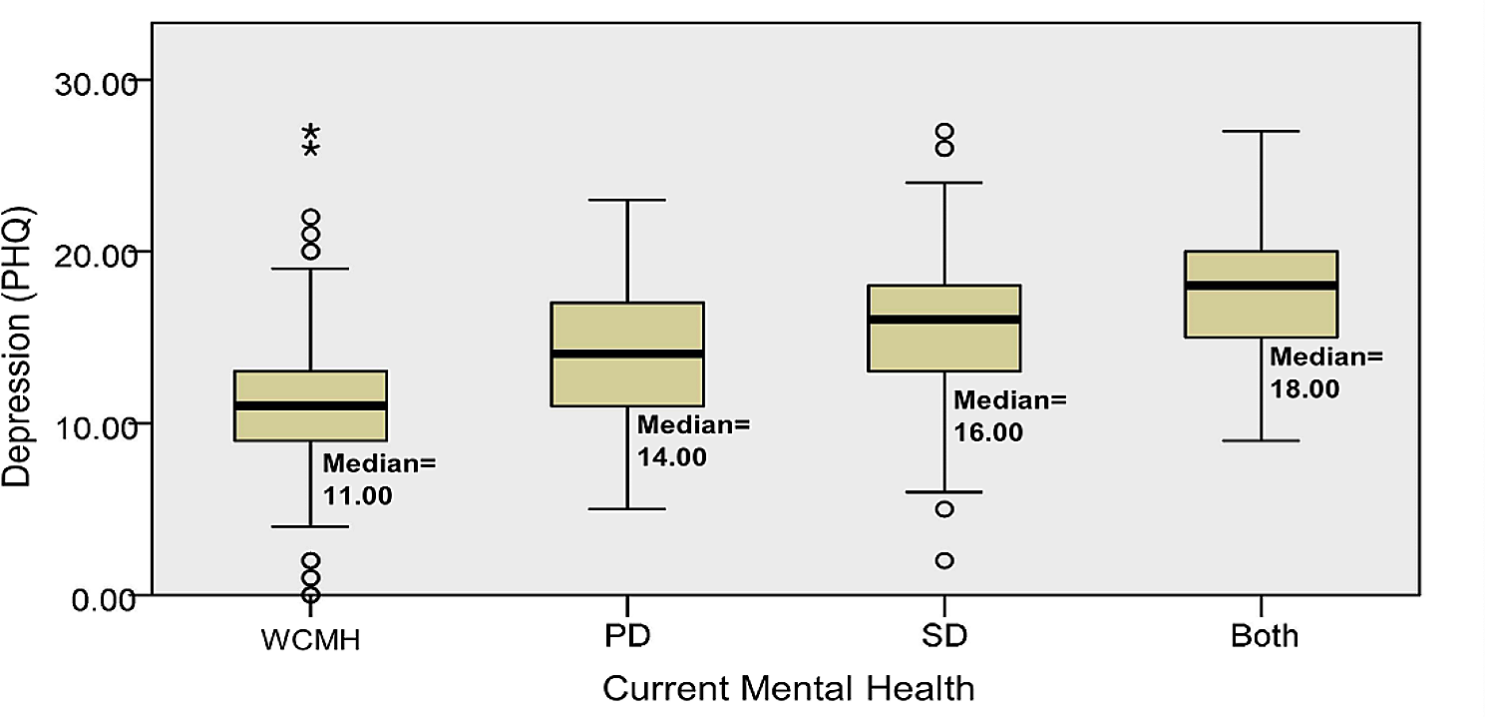
The median difference in depression severity across the four groups of participants. WCMH = without current mental health disturbances, PD = psychological distress, SD = social dysfunction, Both = with psychological distress and social dysfunction

**Fig. 2:**
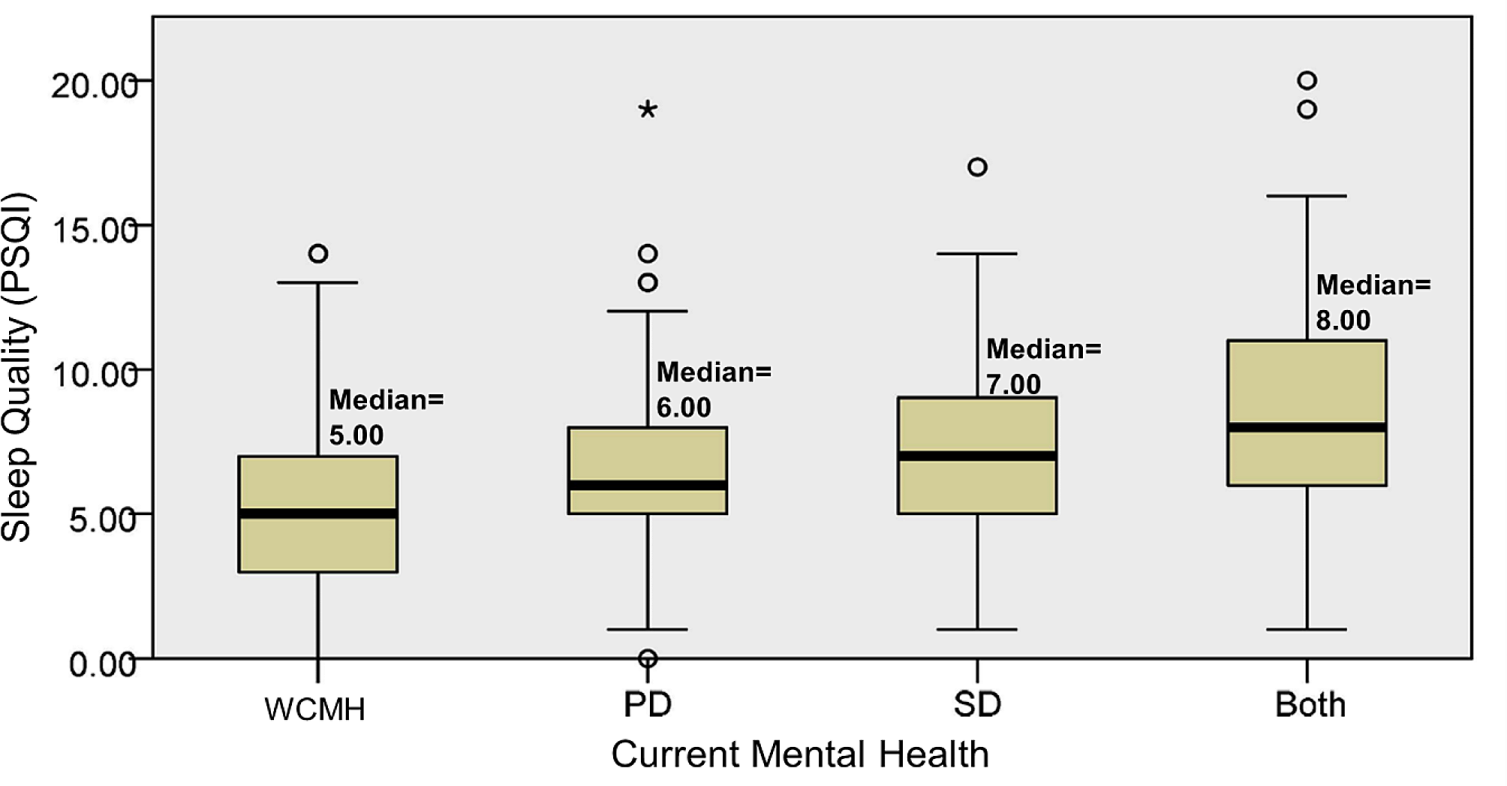
The median difference of sleep quality across the four groups of participants. WCMH = without current mental health disturbances, PD = psychological distress, SD = social dysfunction, Both = with psychological distress and social dysfunction

**Fig. 3:**
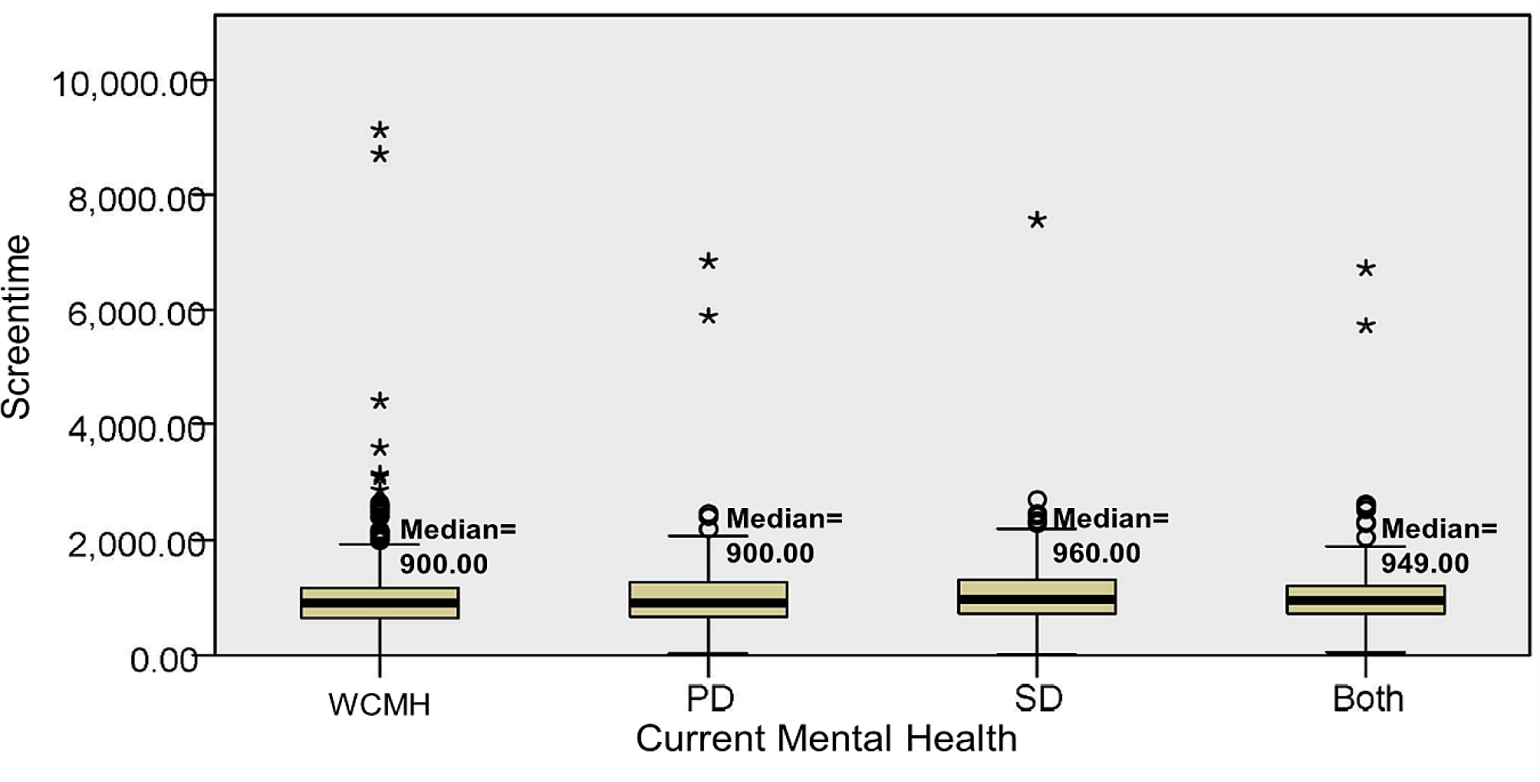
The median difference in screen time across the four groups of participants. WCMH = without current mental health disturbances, PD = psychological distress, SD = social dysfunction, Both = with psychological distress and social dysfunction

**Fig. 4:**
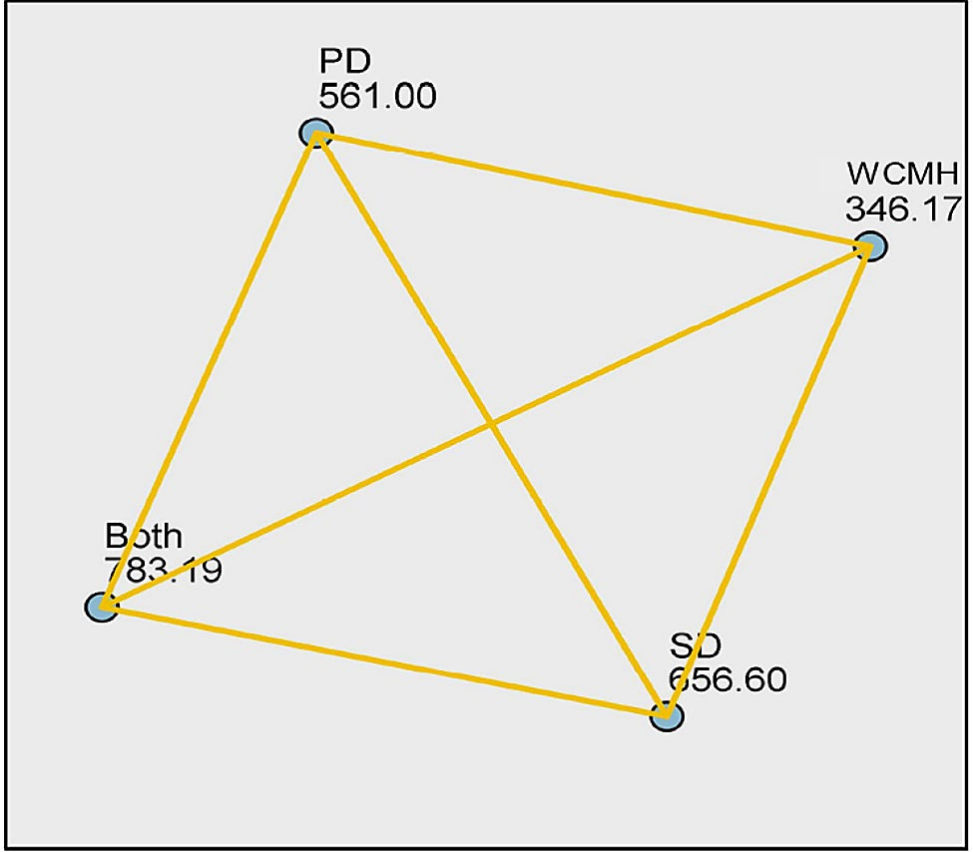
The depression mean rank difference across the four groups of participants. WCMH = without current mental health disturbances, PD = psychological distress, SD = social dysfunction, both = with psychological distress and social dysfunction. Yellow lines indicate statistically significant mean rank differences (*p* < 0.05)

**Fig. 5:**
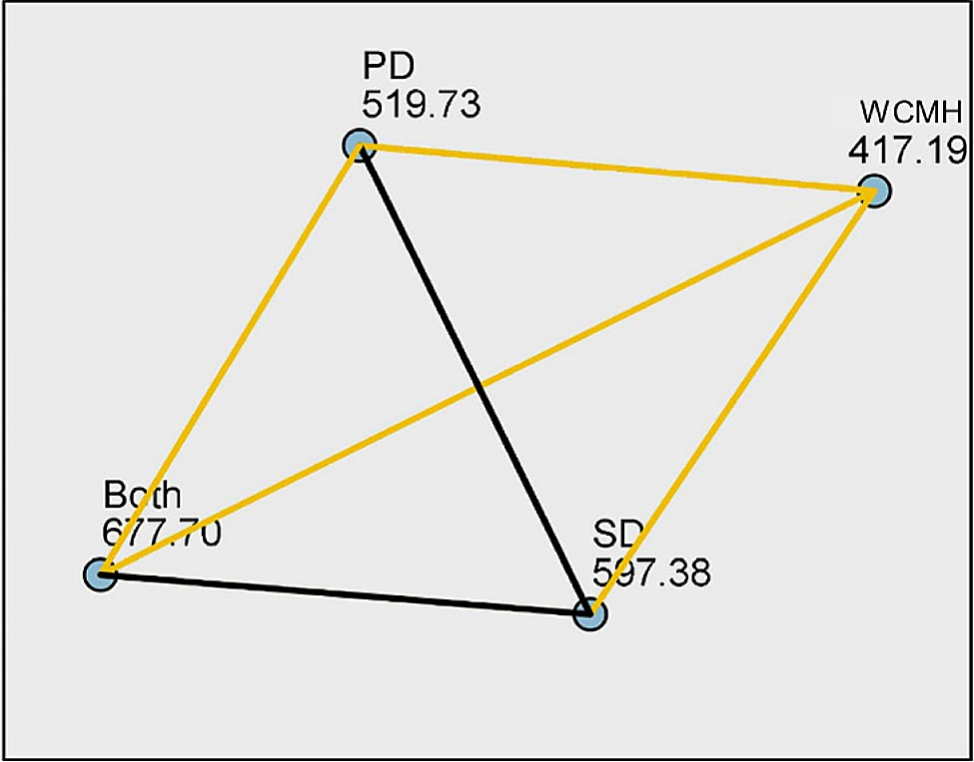
The sleep quality mean rank difference across the four group of participants. WCMH = without current mental health disturbances, PD = psychological distress, SD = social dysfunction, both = with psychological distress and social dysfunction. Yellow lines indicate statistically significant mean rank differences (*p* < 0.05), while black lines indicate a lack of significance (*p* > 0.05)

Statistical analysis demonstrated significant median differences in at least one group pair between depression (*p* < 0.001) and sleep quality (*p* < 0.001) among preclinical medical students without current mental health disturbances, with psychological distress, social dysfunction, or both psychological distress and social dysfunction (Figs. 1 and 2); however, the differences for screen time were not statistically significant (*p* = 0.151) (Fig. 3). The Dunn post-hoc analysis revealed that preclinical medical students without current mental health disturbances had the lowest mean rank of depression and sleep quality compared to the groups with psychological distress, social dysfunction, and having both psychological distress and social dysfunction (*p* < 0.001) (Tables 2 and 3). Meanwhile, preclinical medical students with both psychological distress and social dysfunction displayed the highest mean rank of depression and sleep quality compared to those with only psychological distress or social dysfunction (Figs. 4 and 5).

## Discussion

Mental health is an indisputably important aspect of health and quality of life. For preclinical medical students, the impact of mental health disturbances might manifest more extensively, especially since they are less likely to access care due to worry about stigma and obstacles to career advancement [33]. More broadly, mental health problems could also result in poor outcomes for future patient care, as they disrupt productivity and knowledge attainment [34].

The proportion of current mental health disturbances found in this study, at 49.1%, was comparable to that in previous studies. In Jordanian medical schools, half of the participants had severe mental disorders, and only 13.2% were likely to be well (26); In Turkey, among 1375 medical students, 52.4% of them reported feeling mentally unwell (27); and a study in India showed that nearly half of 2,021 medical students reported some degree of mental health problems, such as depression (50.27%), anxiety (51.46%), and stress (41.61%) [35]. Meanwhile, most students in this study had a moderate degree of depression (35.3%), followed by moderately severe depression (31.7%). In contrast, mild depression was the most prevalent degree in studies conducted in the United States (32.5%) [36] and China (16.98%) [37] using the same research instrument. Females were also shown to be more prone to depression, and freshmen (preclinical students) were more prone than seniors (clinical students) [36]. The majority of respondents in this study were female, thereby possibly contributing to a higher proportion of depression. The influence of current mental health on depression found in this study was straightforward, owing to individual coping mechanisms and characteristics. Nishi et al. [38] previously identified shared factors, such as lack of employment, insufficient social support, and presence of diseases—all mirroring the COVID-19 situation—both in participants with psychological distress only and those with concurrent major depressive disorder. Overall, these findings strengthened the evidence that mental health and depression are inextricably linked and that disturbances in current mental health might explain differences in depression levels.

The current study showed that both psychological distress and social dysfunction were associated with more severe depression and poorer sleep quality. Psychological distress is assumed to be a specific uncomfortable feeling and emotional harm, especially in response to an exclusive stressor or condition, affecting a person temporarily or permanently. For example, a person might be unable to effectively manage stressors, triggering emotional chaos and affecting their physical and mental health [39]. For those diagnosed with psychological distress, a previous study corroborated that worry about being infected with COVID-19 was significantly higher [40]. The level of COVID-19 worry might also be associated with the extent of exposure to information, personal mental health awareness, and levels of depression [41, 42]. In addition, pairwise comparisons in the current study demonstrated higher depression scores and poorer sleep quality in preclinical students with social dysfunction than in those who experienced psychological distress. Social dysfunction refers to operational products that do not function appropriately toward an organized and healthy society within the context of local culture, morals, and values [39]. Given social dysfunction, a social order compatible with the expectations of others and the entire environment cannot be formed [43]. Porcelli et al. explained that social dysfunction is associated with more severe depression and worse sleep quality, in line with the findings in this study [44]. Overall, it could be said that social dysfunction has complex phenotypes that are clearly associated with impairments in social cognition (i.e. the collective mental operations that underlie social interactions, including perceiving, interpreting, and generating responses to the intentions, dispositions, and behaviors of others) [45]. The findings of this study revealed that social relationships were very important for preclinical medical students. During the study period, the COVID-19 pandemic was still in the active phase and the limitation of their social connectivity seemed to be a major problem associated with depression and sleep disturbances.

Overall sleep quality was significantly different between those with distinct states of current mental health disturbances (*p* < 0.05). The overlap between sleep and neurophysiological pathways was reinforced by findings from other studies, demonstrating the bidirectional involvement of sleep as a symptom of psychopathology, as well as the mechanism behind psychological disturbances and social dysfunction [46]. In particular, hyperactivity of the putamen and ventral tegmental areas of the brain in response to stimulation [47], elevated inflammatory markers (IL-6) in response to stressors [48], and disorganized regulation of the cortisol axis during psychological distress– even more so in people with social dysfunction [49]– could all lead to diminished sleep quality. A previous study by Wang et al. [50] found that poor sleep quality was associated with higher mental health disturbances during the COVID-19 pandemic. In this study, no significant difference was found in sleep duration. In previous studies, sleep domain outcomes in correlation with mental health disruptions as measured by the PSQI were not thoroughly consistent. Carbonell et al. [51] found that all domains of sleep quality– especially subjective sleep quality and sleep disturbance, but not sleep latency,– were associated with current mental health disturbances. These findings suggested that current mental health disturbances might be more likely to affect overall sleep quality than individual, variable sleep components.

The statistical analysis revealed that screen time was not different between those with and without current mental health disturbances (*p* = 0.137). Interestingly, other studies discordantly suggested that screen time may be a coping mechanism in times of distress, resulting in an elevated frequency of smartphone addiction in females and games addiction in males [52]. In contrast with previous analyses, the differing measurement and wide-ranging screen time in this study could explain the lack of significance.

Considering that the presence or absence of current mental health disturbances could have considerable implications for daily quality-of-life indicators during COVID-19– particularly for depression and poor sleep quality– greater attention should be given to improve the mental well-being of highly vulnerable preclinical medical students. Medical education institutions should consistently pursue engagement programs that integrate mindfulness therapy, support groups, stress management, and skills training for the mental well-being of students. Stigmas about struggles in the medical profession should be curtailed by promoting transparency in sharing [53]. These efforts are worth pursuing via remote methods, especially during a pandemic, such as COVID-19. Furthermore, during the current postpandemic period, these interventions could also be achieved in person, allowing better advocacy for the mental health of the students. In addition to the main goal of mental well-being itself, sleep and depression could be rectified, especially since these were found to be associated with each other. However, the effectiveness of these various interventions warrant further studies after the pandemic.

Several limitations exist within this study. As the questionnaire was spread online, medical students who did not have access to the Internet or social media groups were excluded. The data were collected over an eight-month period with two waves of COVID-19 peaks, which could result in collection bias due to various changes in the community contexts and government policies. Furthermore, due to the nature of self-reporting measures, social desirability bias could affect the outcomes. Further exploration of specific factors, as well as the effectiveness of mental health intervention strategies for preclinical medical students, would improve the significance of this research.

## Conclusion

Findings from the current study augmented previous evidence by revealing the association between current mental health disturbances and depression, sleep quality, and screen time in Indonesian preclinical medical students. With depression and poor sleep quality, mental health groups yielded exceptionally significant values. In particular, the group with both psychological distress and social dysfunction had worse depression and sleep disturbance than those with either or neither. Thus, in order to increase quality-of-life and success in professional pursuits, medical education intervention strategies should be advocate mental well-being as well as its associated parameters.

## Data Availability

The datasets used and/or analyzed during the current study are available from the corresponding author upon reasonable request.

## Abbreviations

COVID-19: Coronavirus disease 2019
GHQ-12: General Health Questionnaire-12
PHQ-9: Patient Health Questionnaire-9
PSQI: Pittsburgh Sleep Quality Index
SPSS: Statistical Package for Social Sciences
WHO: World Health Organization
sgCC: subgenual cingulate cortex
HPA: hypothalamic‒pituitary‒adrenal
WCMH: without current mental health disturbances
PD: psychological distress
SD: social dysfunction
Both: with psychological distress and social dysfunction

## References

[1] Mental health.: strengthening our response. https://www.who.int/news-room/fact-sheets/detail/mental-health-strengthening-our-response. Accessed 30 Jan 2021.

[2] WHO Coronavirus (COVID-19). Dashboard. https://covid19.who.int. Accessed 4 Jul 2022.

[3] Penninx BWJH, Benros ME, Klein RS, Vinkers CH (2022). How COVID-19 shaped mental health: from infection to pandemic effects. Nat Med.

[4] COVID-19 WRP. Situasi COVID-19 di Indonesia (Update per 4. Juli 2022). covid19.go.id. https://covid19.go.id/artikel/2022/07/04/situasi-covid-19-di-indonesia-update-4-juli-2022. Accessed 4 Jul 2022.

[5] Purborini N, Lee M-B, Devi HM, Chang H-J (2021). Associated factors of depression among young adults in Indonesia: a population-based longitudinal study. J Formos Med Assoc.

[6] Megatsari H, Laksono AD, Ibad M, Herwanto YT, Sarweni KP, Geno RAP et al. The community psychosocial burden during the COVID-19 pandemic in Indonesia. Heliyon. 2020;6. 10.1016/j.heliyon.2020.e05136 PMC7526603 33020744

[7] Chandratre S (2020). Medical students and COVID-19: challenges and supportive strategies. J Med Educ Curric Dev.

[8] Zeng W, Chen R, Wang X, Zhang Q, Deng W (2019). Prevalence of mental health problems among medical students in China: a meta-analysis. Med (Baltim).

[9] Son C, Hegde S, Smith A, Wang X, Sasangohar F (2020). Effects of COVID-19 on College Students’ Mental Health in the United States: interview survey study. J Med Internet Res.

[10] Cuttilan AN, Sayampanathan AA, Ho RC-M (2016). Mental health issues amongst medical students in Asia: a systematic review [2000–2015]. Ann Transl Med.

[11] Nayan MIH, Uddin MSG, Hossain MI, Alam MM, Zinnia MA, Haq I (2022). Comparison of the performance of machine learning-based algorithms for predicting depression and anxiety among University students in Bangladesh: a result of the first wave of the COVID-19 pandemic. Asian J Social Health Behav.

[12] Sharma R, Bansal P, Chhabra M, Bansal C, Arora M (2021). Severe acute respiratory syndrome coronavirus-2-associated perceived stress and anxiety among Indian medical students: a cross-sectional study. Asian J Social Health Behav.

[13] Rotenstein LS, Ramos MA, Torre M, Segal JB, Peluso MJ, Guille C (2016). Prevalence of Depression, depressive symptoms, and suicidal ideation among medical students: a systematic review and Meta-analysis. JAMA.

[14] Marin MG, Nuñez X, de Almeida RMM (2021). Internet addiction and attention in adolescents: a systematic review. Cyberpsychol Behav Soc Netw.

[15] Digital Around the World — DataReportal.– Global Digital Insights. https://datareportal.com/global-digital-overview. Accessed 2 Dec 2022.

[16] Oluwole LO, Obadeji A, Dada MU (2021). Surfing over masked distress: internet addiction and psychological well-being among a population of medical students. Asian J Social Health Behav.

[17] Ranjan LK, Gupta PR, Srivastava M, Gujar NM (2021). Problematic internet use and its association with anxiety among undergraduate students. Asian J Social Health Behav.

[18] Substance use and abuse., COVID-19-related distress, and disregard for social distancing: A network analysis - PubMed. https://pubmed.ncbi.nlm.nih.gov/33310690/. Accessed 7 Jul 2022. 10.1016/j.addbeh.2020.106754 PMC8164919 33310690

[19] Joensen LE, Steenberg JL, Madsen KP, Willaing I. What people with diabetes in Denmark worry about during the COVID-19 pandemic: a longitudinal study of the first 3 months of the COVID-19 pandemic. Diabetic Med. 2021;38. 10.1111/dme.14665 PMC8420213 34327749

[20] Barthorpe A, Winstone L, Mars B, Moran P (2020). Is social media screen time really associated with poor adolescent mental health? A time use diary study. J Affect Disord.

[21] Huckins JF, daSilva AW, Wang R, Wang W, Hedlund EL, Murphy EI (2019). Fusing Mobile phone sensing and Brain Imaging to Assess Depression in College Students. Front Neurosci.

[22] Freeman D, Sheaves B, Waite F, Harvey AG, Harrison PJ. Sleep disturbance and psychiatric disorders. The lancet. Psychiatry. 2020;7. https://pubmed.ncbi.nlm.nih.gov/32563308/. Accessed 30 Jan 2021. 10.1016/S2215-0366(20)30136-X 32563308

[23] Malhi GS, Mann JJ, Depression. The Lancet. 2018;392:2299–312. 10.1016/S0140-6736(18)31948-2 30396512

[24] Al-Khani AM, Sarhandi MI, Zaghloul MS, Ewid M, Saquib N (2019). A cross-sectional survey on sleep quality, mental health, and academic performance among medical students in Saudi Arabia. BMC Res Notes.

[25] Roberts RE, Duong HT (2014). The prospective association between sleep deprivation and depression among adolescents. Sleep.

[26] Idaiani S, Suhardi S. Validitas Dan Reliabilitas General Health Questionnaire untuk skrining distres psikologik dan disfungsi sosial di masyarakat. Buletin Penelitian Kesehatan. 2006;34 4 Des.

[27] Van der Linden A. Cross-cultural validation of the Patient Health Questionnaire (PHQ-9) in Bahasa Indonesia to measure depression among people affected by leprosy in Central Java, Indonesia. Vrije Universiteit Amsterdam; 2019.

[28] Kroenke K, Spitzer RL, Williams JB (2001). The PHQ-9: validity of a brief depression severity measure. J Gen Intern Med.

[29] Indrawati N. Perbandingan Kualitas Tidur Mahasiswa Yang mengikuti UKM Dan Tidak Mengikuti UKM pada mahasiswa reguler FIK UI. Universitas Indonesia; 2012.

[30] Buysse DJ, Reynords CF 3rd, Monk TH, Berman SR, Kupfer DJ. The Pittsburgh Sleep Quality Index: a new instrument for psychiatric practice and research. Psychiatry research. 1989;28. https://pubmed.ncbi.nlm.nih.gov/2748771/. Accessed 30 Jan 2021. 10.1016/0165-1781(89)90047-4 2748771

[31] Klakk H, Wester CT, Olesen LG, Rasmussen MG, Kristensen PL, Pedersen J (2020). The development of a questionnaire to assess leisure time screen-based media use and its proximal correlates in children (SCREENS-Q). BMC Public Health.

[32] Lakens D. Calculating and reporting effect sizes to facilitate cumulative science: a practical primer for t-tests and ANOVAs. Front Psychol. 2013;4. 10.3389/fpsyg.2013.00863 PMC3840331 24324449

[33] Jacob R, Li T, Martin Z, Burren A, Watson P, Kant R (2020). Taking care of our future doctors: a service evaluation of a medical student mental health service. BMC Med Educ.

[34] Azim SR. Mental distress among medical students. IntechOpen; 2020.

[35] Jiang D, Chen J, Liu Y, Lin J, Liu K, Chen H (2021). Patterns of mental health problems before and after easing COVID-19 restrictions: evidence from a 105248-subject survey in general population in China. PLoS ONE.

[36] Wang X, Hegde S, Son C, Keller B, Smith A, Sasangohar F (2020). Investigating Mental Health of US College Students during the COVID-19 Pandemic: cross-sectional survey study. J Med Internet Res.

[37] Chang J, Yuan Y, Wang D (2020). [Mental health status and its influencing factors among college students during the epidemic of COVID-19]. Nan Fang Yi Ke Da Xue Xue Bao.

[38] Nishi D, Imamura K, Watanabe K, Ishikawa H, Tachimori H, Takeshima T (2020). Psychological distress with and without a history of depression: results from the World Mental Health Japan 2nd Survey (WMHJ2). J Affect Disord.

[39] Sahu PK, Nayak BS, Rodrigues V, Umakanthan S (2020). Prevalence of psychological distress among Undergraduate Medical students: a cross-sectional study. Int J Appl Basic Med Res.

[40] Samuels J, Holingue C, Nestadt PS, Bienvenu OJ, Phan P, Nestadt G (2021). An investigation of COVID-19 related worry in a United States population sample. J Psychiatr Res.

[41] Meltzer GY, Chang VW, Lieff SA, Grivel MM, Yang LH, Des Jarlais DC (2021). Behavioral correlates of COVID-19 worry: Stigma, Knowledge, and News source. Int J Environ Res Public Health.

[42] Wahlund T, Mataix-Cols D, Olofsdotter Lauri K, de Schipper E, Ljótsson B, Aspvall K (2021). Brief online cognitive behavioural intervention for dysfunctional worry related to the COVID-19 pandemic: a Randomised Controlled Trial. Psychother Psychosom.

[43] van der Wee NJA, Bilderbeck AC, Cabello M, Ayuso-Mateos JL, Saris S, Giltay EJ et al. Working definitions, subjective and objective assessments and experimental paradigms in a study exploring social withdrawal in schizophrenia and Alzheimer’s disease. Neurosci Biobehav Rev. 2019;97. 10.1016/j.neubiorev.2018.06.020 29949732

[44] Porcelli S, Van der Wee N, van der Werff S, Aghajani M, Glennon G, van Heukelum S et al. Social brain, social dysfunction and social withdrawal. Neurosci Biobehav Rev. 2019;97. 10.1016/j.neubiorev.2018.09.012 30244163

[45] Gur RC, Gur RE (2016). Social cognition as an RDoC domain. Am J Med Genet Part B: Neuropsychiatric Genet.

[46] Palmer CA, Alfano CA. Sleep and emotion regulation: An organizing, integrative review. Sleep medicine reviews. 2017;31. https://pubmed.ncbi.nlm.nih.gov/26899742/. Accessed 30 Jan 2021. 10.1016/j.smrv.2015.12.006 26899742

[47] Gujar N, Yoo S-S, Hu P, Walker MP. Sleep deprivation amplifies reactivity of brain reward networks, biasing the appraisal of positive emotional experiences. The Journal of neuroscience: the official journal of the Society for Neuroscience. 2011;31. https://pubmed.ncbi.nlm.nih.gov/21430147/. Accessed 30 Jan 2021. 10.1523/JNEUROSCI.3220-10.2011 PMC3086142 21430147

[48] Heffner KL, Ng HM, Suhr JA, France CR, Marshall GD, Pigeon WR et al. Sleep disturbance and older adults’ inflammatory responses to acute stress. The American journal of geriatric psychiatry: official journal of the American Association for Geriatric Psychiatry. 2012;20. https://pubmed.ncbi.nlm.nih.gov/22327621/. Accessed 30 Jan 2021. 10.1097/JGP.0b013e31824361de PMC3588882 22327621

[49] Minkel J, Moreta M, Muto J, Htaik O, Jones C, Basner M et al. Sleep deprivation potentiates HPA axis stress reactivity in healthy adults. Health psychology: official journal of the Division of Health Psychology, American Psychological Association. 2014;33. https://pubmed.ncbi.nlm.nih.gov/24818608/. Accessed 30 Jan 2021. 10.1037/a0034219 24818608

[50] Wang D, Ross B, Zhou X, Meng D, Zhu Z, Zhao J (2021). Sleep disturbance predicts suicidal ideation during COVID-19 pandemic: a two-wave longitudinal survey. J Psychiatr Res.

[51] Clement-Carbonell V, Portilla-Tamarit I, Rubio-Aparicio M, Madrid-Valero JJ (2021). Sleep Quality, Mental and Physical Health: a Differential Relationship. Int J Environ Res Public Health.

[52] Chen B, Liu F, Ding S, Ying X, Wang L, Wen Y (2017). Gender differences in factors associated with smartphone addiction: a cross-sectional study among medical college students. BMC Psychiatry.

[53] Tian-Ci Quek T, Wai-San Tam W, Tran X, Zhang B, Zhang M, Su-Hui Ho Z (2019). The global prevalence of anxiety among medical students: a Meta-analysis. Int J Environ Res Public Health.

